# Gut dysbiosis and mortality in hemodialysis patients

**DOI:** 10.1038/s41522-021-00191-x

**Published:** 2021-03-03

**Authors:** Ting-Yun Lin, Ping-Hsun Wu, Yi-Ting Lin, Szu-Chun Hung

**Affiliations:** 1 Division of Nephrology, Taipei Tzu Chi Hospital, Buddhist Tzu Chi Medical Foundation and School of Medicine, Tzu Chi University, Hualien, Taiwan; 2 Division of Nephrology, Department of Internal Medicine, Kaohsiung Medical University Hospital, Kaohsiung Medical University, Kaohsiung, Taiwan; 3 Faculty of Medicine, College of Medicine, Kaohsiung Medical University, Kaohsiung, Taiwan; 4 Department of Family Medicine, Kaohsiung Medical University Hospital, Kaohsiung Medical University, Kaohsiung, Taiwan

**Keywords:** Applied microbiology, Clinical microbiology

## Abstract

Little is known about the relationship between gut dysbiosis, inflammation, and adverse outcomes in patients with chronic kidney disease. We examined the association of microbial diversity with all-cause mortality in hemodialysis patients. The gut microbiota was assessed by 16S ribosomal RNA gene sequencing. During a median follow-up of 2.1 years, the adjusted risk of death among patients with higher diversity (above median) was 74% lower than that among patients with lower diversity (below median). We then compared the microbial composition between nonsurvivors and survivors in a matched case-control study. We observed significantly lower microbial diversity and higher proinflammatory cytokines among nonsurvivors than survivors. Specifically, the relative abundance of *Succinivibrio* and *Anaerostipes*, two short-chain fatty acid-producing bacteria, was markedly reduced in nonsurvivors. Thus, a unique gut microbial composition is associated with an increased risk of mortality among hemodialysis patients and may be used to identify subjects with a poor prognosis.

## Introduction

Patients with end-stage kidney disease (ESKD) receiving dialysis have a significantly reduced life expectancy compared to the general population^1^. Both cardiovascular (CV) and non-CV mortality risks are equally increased^2^. Identifying factors that are associated with this higher risk is important in the care of patients with ESKD. Observational studies among patients on dialysis have pointed out several predictors for the greater mortality risk, including older age and the high prevalence of comorbidities, especially diabetes mellitus (DM) and cardiovascular diseases (CVD) (traditional risk factors)^3^, as well as malnutrition, inflammation, and accumulation of uremic solutes (nontraditional risk factors)^4–6^.

A growing number of studies have shown that the gut microbiota is crucial for protecting against pathogens and maintaining normal immune and metabolic homeostasis^7^. The healthy human gastrointestinal tract harbors a highly diverse population of microorganisms. In contrast, uremia alters the normal composition and function of the gut microbiota, commonly referred to as gut dysbiosis^8^. Gut dysbiosis promotes local and systemic inflammation that leads to various pathological consequences. Accumulating evidence also indicates that gut-derived uremic toxins are involved in the pathogenesis of CVD in ESKD^9^.

Gut dysbiosis is characterized by loss of diversity and imbalance in composition. High α-diversity, a measure of bacterial richness and evenness, is often associated with better health status^10^. The association of lower microbial diversity with poor survival has been described in patients undergoing allogeneic hematopoietic-cell transplantation and in patients hospitalized for chronic obstructive pulmonary disease^11,12^. We have recently demonstrated that malnutrition and inflammation are correlated with a significant decrease in gut microbial diversity in patients with ESKD^13,14^. However, it is unclear whether decreased gut microbial diversity is associated with adverse outcomes in ESKD. The aim of this study was to explore whether gut dysbiosis can predict the risk of death in an observational ESKD cohort. Furthermore, we examined the gut microbiota profile among nonsurvivors and survivors in a matched case-control study.

## Results

### Patient characteristics

**Fig. 1:**
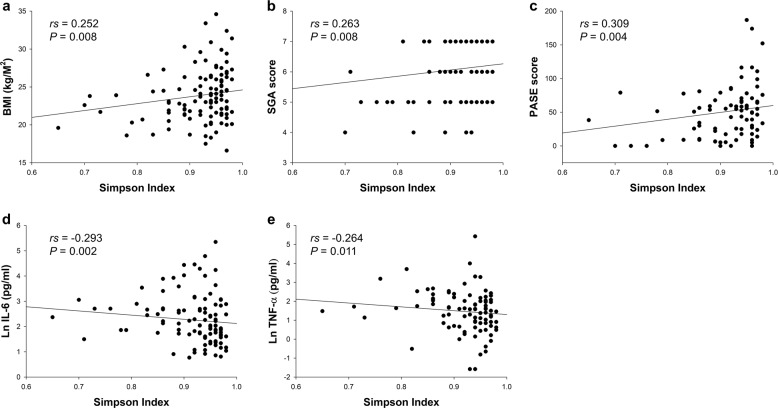
Factors associated with the Simpson index. Univariate analysis of the correlation of the Simpson index with **a** BMI, **b** SGA score, **c** PASE score, **d** IL-6, and **e** TNF-α. Natural logarithmic transformation of IL-6 and TNF-α was used to normalize the distributions for univariate analyses. BMI body mass index, IL-6 interleukin-6, PASE the Physical Activity Scale for the Elderly, SGA subjective global assessment, TNF-α tumor necrosis factor-α.

A total of 109 patients were enrolled in the study. Overall, the mean age was 68.4 ± 10.4 years, with 57 men and 52 women; 49.5% had DM (*n* = 54), and 45.9% had CVD (*n* = 50). The dialysis vintage was 8.0 (4.6–11.0) years. Across the 109 fecal samples, the total number of merged reads was 17,520,918, and after filtering steps, 11,968,852 reads were considered for analysis. Patients were stratified into higher-diversity and lower-diversity groups by the median Simpson index to evaluate the association between gut microbial diversity and mortality. We found that patients with lower diversity were more likely to have a higher prevalence of CVD and other comorbidities and had a significantly lower subjective global assessment (SGA) score, Physical Activity Scale for the Elderly (PASE) score, and plasma intact parathyroid hormone but significantly higher inflammatory markers, including interleukin-6 (IL-6) and tumor necrosis factor-α (TNF-α) (Table 1). Microbial diversity, expressed as the Simpson index, was significantly correlated with several baseline variables, including body mass index (BMI) (*rs* = 0.252, *P* = 0.008), SGA score (*rs* = 0.263 *P* = 0.008), PASE score (*rs* = 0.309, *P* = 0.004), IL-6 (*rs* = −0.293, *P* = 0.002), and TNF-α (*rs* = −0.264, *P* = 0.011) (Fig. 1). The correlations remained significant after adjustment for age, sex, and the Charlson comorbidity index, as determined by Spearman’s partial correlation analyses. The gut microbial composition among patients with higher and lower microbial diversity at the phylum and genus levels are shown in Supplementary Fig. 1.

**Table 1:** Baseline characteristics of patients according to the median Simpson index.

Parameter	Lower diversity (*n* = 54)	Higher diversity (*n* = 55)	*P* Value
Age (years)	69 ± 11	68 ± 10	0.486
Male sex, *n* (%)	32 (59.3%)	25 (45.5%)	0.149
DM, *n* (%)	29 (53.7%)	25 (45.5%)	0.389
CVD, *n* (%)	32 (59.3%)	18 (32.7%)	0.005
Charlson comorbidity index			
1–2	1 (1.9%)	0 (0.0%)	
3–4	5 (9.3%)	15 (27.3%)^a^	0.035
≥5	48 (88.9%)	40 (72.7%)^a^	
Dialysis vintage (years)	7.5 (4.8–12.0)	8.0 (4.0–11.0)	0.752
Dialysis access type			
Arteriovenous fistula	47 (87.0%)	49 (89.1%)	
Arteriovenous graft	6 (11.1%)	6 (10.9%)	0.597
Central venous catheter	1 (1.9%)	0 (0.0%)	
BMI (kg/m^2^)	23.2 ± 3.5	24.5 ± 3.4	0.053
FTI (kg/m^2^)	8.9 ± 4.1	10.2 ± 3.9	0.098
LTI (kg/m^2^)	13.3 ± 3.6	13.7 ± 2.7	0.596
BF (%)	27.8 ± 10.9	30.1 ± 9.3	0.230
Overhydration (%)	4.1 (−3.2–9.7)	4.5 (−2.5–10.9)	0.445
SGA score	6.0 (5.0–7.0)	6.0 (6.0–7.0)	0.007
PASE score	32 (9–59)	58 (35–86)	0.002
Medication			
PPI, *n* (%)	5 (9.4%)	5 (9.1%)	0.951
Calcium carbonate, *n* (%)	40 (75.5%)	49 (89.1%)	0.063
CCB, *n* (%)	26 (49.1%)	25 (45.5%)	0.708
β-blocker, *n* (%)	24 (45.3%)	27 (49.1%)	0.692
RAASi, *n* (%)	20 (37.7%)	16 (29.1%)	0.341
Dietary intake (servings/day)			
Vegetables	2.4 (1.5–3.3)	2.2 (1.6–3.1)	0.867
Fruits	0.9 (0.3–1.1)	0.6 (0.3–1.4)	0.904
Meat	0.9 (0.4–1.3)	0.7 (0.4–1.3)	0.699
Laboratory parameters			
Kt/V	1.7 ± 0.4	1.7 ± 0.3	0.740
nPNA (g/kg/day)	1.2 ± 0.2	1.1 ± 0.3	0.755
Albumin (g/dl)	3.9 ± 0.3	4.0 ± 0.4	0.536
Fasting glucose (mg/dl)	115 (96–150)	109 (100–140)	0.587
Total cholesterol (mg/dl)	153 (136–191)	157 (138–179)	0.901
LDL (mg/dl)	86 (66–106)	90 (73–106)	0.248
Triglycerides (mg/dl)	102 (70–177)	101 (70–151)	0.513
Calcium (mmol/l)	9.3 ± 0.8	9.3 ± 0.9	0.753
Phosphorus (mg/dl)	5.1 ± 1.4	5.3 ± 1.2	0.296
iPTH (pg/ml)	257 (107–425)	398 (187–605)	0.034
CRP (mg/dl)	0.32 (0.17–0.85)	0.23 (0.14–0.61)	0.154
IL-6 (pg/ml)	12.38 (6.35–18.91)	6.46 (4.07–11.83)	0.002
TNF-α (pg/ml)	5.02 (1.86–10.76)	2.57 (0.68–5.89)	0.009

*BF* body fat, *BMI* body mass index, *CCB* calcium channel blocker, *CRP* C-reactive protein, *CVD* cardiovascular disease*, DM* diabetes mellitus, *FTI* fat tissue index, *IL-6* interleukin-6, *iPTH* intact parathyroid hormone*, LDL* low-density lipoprotein, *LTI* lean tissue index, *nPNA* normalized protein nitrogen appearance, *PASE* the Physical Activity Scale for the Elderly, *PPI* proton pump inhibitor, *RAASi* renin-angiotensin-aldosterone system inhibitor, *SGA* subjective global assessment; *TNF-α* tumor necrosis factor-α.

^a^*P* < 0.05 between the lower-diversity and the higher-diversity group by the Bonferroni post hoc test.

### Association between microbial diversity and survival

**Fig. 2:**
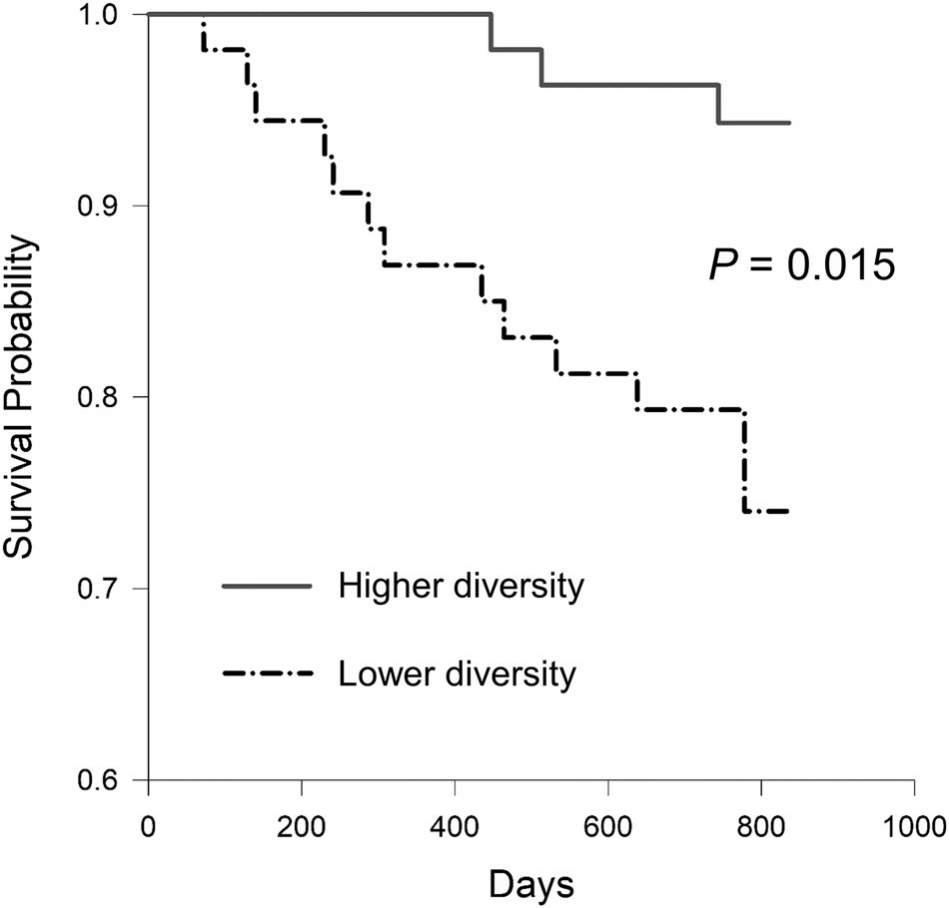
Kaplan–Meier analysis curves. Hemodialysis patients were stratified by the median of the Simpson index to assess the unadjusted risks for all-cause mortality.

During a median follow-up of 2.1 years, 15 (13.8%) patients died. The majority of deaths were due to non-CV causes (*n* = 11), whereas 4 patients died of CVD. The most common causes of non-CV death were infections (*n* = 5) and malignancies (*n* = 2). Kaplan–Meier analysis showed that the risk of death was significantly greater in patients with lower diversity (*n* = 54) than in those with higher diversity (*n* = 55) (*P* = 0.015) (Fig. 2). The estimated overall survival was 94.5% for the higher-diversity group and 77.8% for the lower-diversity group. Cox proportional hazards analyses for overall survival are shown in Table 2. Microbial diversity at baseline was significantly associated with the risk of death from any cause. Specifically, patients with higher diversity had 74% risk reduction for mortality compared with their lower-diversity counterparts in age-, sex-, and comorbidity-adjusted models (adjusted hazard ratio, 0.26; 95% confidence interval, 0.07–0.95, *P* = 0.041).

**Table 2:** Multivariable Cox regression analysis for the relative risk of all-cause mortality calculated for a Simpson index below or above the median.

	Unadjusted	Model 1	Model 2
	HR (95% CI)	HR (95% CI)	HR (95% CI)
*Simpson index by median*			
Lower	1.0	1.0	1.0
Higher	0.24 (0.07–0.84)	0.24 (0.07–0.87)	0.26 (0.07–0.95)
*P* value	0.026	0.030	0.041

*CI* confidence interval, *HR* hazard ratio.

Model 1 was adjusted for age and sex.

Model 2 was adjusted for age, sex, and the Charlson comorbidity index.

Additional analyses for microbial diversity with risk of CV events and infection-related hospitalizations are shown in Supplementary Table 1. There were 24 CV events, including nonfatal myocardial infarction (*n* = 10), nonfatal stroke (*n* = 3), hospitalization for congestive heart failure (*n* = 7), and CV death (*n* = 4). Twenty-eight subjects experienced an infection event requiring hospitalization, consisting of pulmonary infection (*n* = 13), genitourinary infection (*n* = 2), gastrointestinal or hepatobiliary infection (*n* = 2), skin and soft tissue infection (*n* = 4), bloodstream infection or sepsis (*n* = 4), and other infections (*n* = 3). We found that higher microbial diversity was associated with a significantly lower risk of CV events (adjusted hazard ratio, 0.36; 95% confidence interval, 0.15–0.88, *P* = 0.026). However, there was no significant association between microbial diversity and the risk of infection (adjusted hazard ratio, 0.71; 95% confidence interval, 0.33–1.52, *P* = 0.374).

### Characteristics of nonsurvivors and survivors

In general, nonsurvivors (*n* = 15) were older than survivors (*n* = 94) (Table 3). Otherwise, the two groups were similar regarding sex, the prevalence of DM and preexisting CVD, dialysis vintage, physical activity, and nutritional status, including the SGA score, BMI, and serum albumin. In addition, there was no significant difference in the use of medications known to affect the gut microbiota, as well as dietary consumption patterns of vegetables, fruits, or meat between the two groups. We observed that nonsurvivors had significantly lower levels of total cholesterol and low-density lipoprotein cholesterol but significantly higher levels of IL-6 and TNF-α than survivors. Moreover, a significantly lower proportion of nonsurvivors used arteriovenous fistula as vascular access compared to survivors.

**Table 3:** Baseline characteristics of patients according to mortality status before matching.

Characteristic	Dead (*n* = 15)	Alive (*n* = 94)	*P* Value
Age (years)	74 ± 10	68 ± 10	0.031
Male sex, *n* (%)	7 (46.7%)	50 (53.2%)	0.638
DM, *n* (%)	6 (40.0%)	48 (51.1%)	0.426
CVD, *n* (%)	9 (60.0%)	41 (43.6%)	0.237
Charlson comorbidity index			
1–2	0 (0.0%)	1 (1.1%)	
3–4	0 (0.0%)	20 (21.3%)	0.126
≥5	15 (100.0%)	73 (77.7%)	
Dialysis vintage (years)	7.0 (6.0–9.0)	8.0 (4.1–11.1)	0.747
Dialysis access type			
Arteriovenous fistula	10 (66.7%)	86 (91.5%)	
Arteriovenous graft	4 (26.7%)	8 (8.5%)	0.004
Central venous catheter	1 (6.7%)	0 (0.0%)	
BMI (kg/m^2^)	22.6 ± 3.3	24.0 ± 3.5	0.146
FTI (kg/m^2^)	8.7 ± 4.7	9.7 ± 3.9	0.363
LTI (kg/m^2^)	13.4 ± 3.0	13.5 ± 3.2	0.886
BF (%)	27.2 ± 12.5	29.2 ± 9.8	0.469
Overhydration (%)	5.8 (−5.4–12.4)	4.1 (−2.6–10.0)	0.829
SGA score	5.5 (5.0–7.0)	6.0 (6.0–7.0)	0.082
PASE score	9 (0–68)	51 (23–67)	0.080
Medication			
PPI, *n* (%)	1 (7.1%)	9 (9.6%)	0.770
Calcium carbonate, *n* (%)	10 (71.4%)	79 (84.0%)	0.248
CCB, *n* (%)	6 (42.9%)	45 (47.9%)	0.726
β-blocker, *n* (%)	7 (50.0%)	44 (46.8%)	0.823
RAASi, *n* (%)	4 (28.6%)	32 (34.0%)	0.685
Dietary intake (servings/day)			
Vegetables	1.8 (0.4–2.7)	2.3 (1.6–3.3)	0.059
Fruits	0.7 (0.2–1.0)	0.7 (0.3–1.2)	0.559
Meat	0.6 (0.0–1.3)	0.7 (0.4–1.3)	0.353
Laboratory parameters			
Kt/V	1.6 ± 0.6	1.8 ± 0.3	0.142
nPNA (g/kg/day)	1.1 ± 0.2	1.2 ± 0.3	0.164
Albumin (g/dl)	3.8 ± 0.3	4.0 ± 0.4	0.148
Fasting glucose (mg/dl)	112 (97–147)	109 (99–147)	0.847
Total cholesterol (mg/dl)	135 (124–152)	158 (143–186)	0.012
LDL (mg/dl)	74 (63–87)	90 (72–111)	0.016
Triglycerides (mg/dl)	88 (58–192)	106 (75–154)	0.305
Calcium (mmol/l)	9.2 ± 0.8	9.3 ± 0.8	0.544
Phosphorus (mg/dl)	4.9 ± 1.5	5.3 ± 1.3	0.389
iPTH (pg/ml)	183 (25–467)	345 (179–517)	0.249
CRP (mg/dl)	0.30 (0.14–0.88)	0.27 (0.15–0.62)	0.556
IL-6 (pg/ml)	16.87 (11.57–34.59)	7.15 (4.90–14.67)	0.002
TNF-α (pg/ml)	7.15 (1.86–18.67)	3.40 (1.27–6.54)	0.040

*BF* body fat, *BMI* body mass index, *CCB* calcium channel blocker, *CRP* C-reactive protein, *CVD* cardiovascular disease, *DM* diabetes mellitus, *FTI* fat tissue index, *IL-6* interleukin-6, *iPTH* intact parathyroid hormone, *LDL* low-density lipoprotein, *LTI* lean tissue index, *nPNA* normalized protein nitrogen appearance, *PASE* the Physical Activity Scale for the Elderly, *PPI* proton pump inhibitor, *RAASi* renin-angiotensin-aldosterone system inhibitor, *SGA* subjective global assessment, *TNF-α* tumor necrosis factor-α.

### Gut microbial composition according to mortality status after matching

We then conducted a case-control study for the comparison of microbial composition according to mortality status. Fourteen nonsurvivors were matched to 56 survivors (1:4 matching) by age (±5 years) and sex according to a prespecified statistical plan (Table 4). One nonsurvivor was excluded from the analysis because there were no available matches from the survivors who satisfied the age matching criteria. The distribution of baseline characteristics before and after matching was relatively consistent among the two groups except for a higher SGA score in survivors.

**Table 4:** Baseline characteristics of patients according to mortality status after matching.

Characteristic	Dead (*n* = 14)	Alive (*n* = 56)	*P* Value
Age (years)	73 ± 9	71 ± 9	0.419
Male sex, *n* (%)	7 (50.0%)	28 (50.0%)	1.000
DM, *n* (%)	6 (42.9%)	32 (57.1%)	0.337
CVD, *n* (%)	9 (64.3%)	28 (50.0%)	0.338
Charlson comorbidity index			
1–2	0 (0.0%)	0 (0.0%)	
3–4	0 (0.0%)	8 (14.3%)	0.133
≥5	14 (100.0%)	48 (85.7%)	
Dialysis vintage (years)	7.0 (5.5–9.8)	8.0 (4.0–11.3)	0.797
Dialysis access type			
Arteriovenous fistula	9 (64.3%)	51 (91.1%)	
Arteriovenous graft	4 (28.6%)	5 (8.9%)	0.015
Central venous catheter	1 (7.1%)	0 (0.0%)	
BMI (kg/m^2^)	22.4 ± 3.3	23.9 ± 3.5	0.134
FTI (kg/m^2^)	8.3 ± 4.5	9.6 ± 4.3	0.300
LTI (kg/m^2^)	13.6 ± 3.1	13.3 ± 3.4	0.813
BF (%)	26.1 ± 12.3	28.9 ± 10.7	0.405
Overhydration (%)	6.6 (−5.1–13.2)	6.1 (−0.9–10.0)	0.912
SGA score	5.0 (5.0–6.5)	6.0 (6.0–7.0)	0.032
PASE score	17 (2–73)	54 (22–71)	0.204
Medication			
PPI, *n* (%)	1 (7.7%)	4 (7.1%)	0.945
Calcium carbonate, *n* (%)	9 (69.2%)	45 (80.4%)	0.381
CCB, *n* (%)	6 (46.2%)	28 (50.0%)	0.803
β-blocker, *n* (%)	7 (53.8%)	26 (46.4%)	0.630
RAASi, *n* (%)	4 (30.8%)	21 (37.5%)	0.649
Dietary intake (servings/day)			
Vegetables	1.9 (0.3–2.7)	2.3 (1.8–3.2)	0.083
Fruits	0.7 (0.2–1.0)	0.6 (0.3–1.2)	0.497
Meat	0.6 (0.0–1.3)	0.7 (0.4–1.2)	0.696
Laboratory parameters			
Kt/V	1.6 ± 0.6	1.8 ± 0.3	0.128
nPNA (g/kg/day)	1.1 ± 0.2	1.2 ± 0.3	0.095
Albumin (g/dl)	3.8 ± 0.3	4.0 ± 0.3	0.202
Fasting glucose (mg/dl)	115 (100–157)	111 (101–156)	0.877
Total cholesterol (mg/dl)	138 (122–155)	157 (144–187)	0.033
LDL (mg/dl)	75 (68–87)	89 (69–111)	0.053
Triglycerides (mg/dl)	89 (59–193)	101 (68–153)	0.681
Calcium (mmol/l)	9.1 ± 0.8	9.4 ± 0.9	0.354
Phosphorus (mg/dl)	4.9 ± 1.5	5.1 ± 1.3	0.503
iPTH (pg/ml)	166 (24–494)	295 (185–577)	0.154
CRP (mg/dl)	0.36 (0.14–1.07)	0.26 (0.15–0.59)	0.436
IL-6 (pg/ml)	17.50 (10.59–34.95)	6.86 (5.06–14.76)	0.006
TNF-α (pg/ml)	7.07 (1.71–24.07)	2.53 (0.50–5.06)	0.026

*BF* body fat, *BMI* body mass index, *CCB* calcium channel blocker, *CRP* C-reactive protein, *CVD* cardiovascular disease, *DM* diabetes mellitus, *FTI* fat tissue index, *IL-6* interleukin-6, *iPTH* intact parathyroid hormone, *LDL* low-density lipoprotein, *LTI* lean tissue index, *nPNA* normalized protein nitrogen appearance, *PASE* the Physical Activity Scale for the Elderly, *PPI* proton pump inhibitor*, RAASi* renin-angiotensin-aldosterone system inhibitor, *SGA* subjective global assessment, *TNF-α* tumor necrosis factor-α.

Overall, the relative abundance of bacterial taxa at the phylum level did not reveal significant differences between the groups. The majority of the taxa (displayed as the median relative abundance) among the two groups belonged to *Bacteroidetes* (59.3% and 61.7% for survivors and nonsurvivors, respectively, *P* = 0.860), followed by *Firmicutes* (26.3% and 23.2% for survivors and nonsurvivors, respectively, *P* = 0.895) and *Proteobacteria* (6.9% and 7.0% for survivors and nonsurvivors, respectively, *P* = 1.000). In addition, the *Firmicutes/Bacteroidetes* ratio did not differ between the two groups (0.45 and 0.35 for survivors and nonsurvivors, respectively, *P* = 0.941). At the genus level, there was no significant difference in distribution according to the enterotypes between the two groups. The *Bacteroides* enterotype was predominant among the two groups (83.9% and 71.4% for survivors and nonsurvivors, respectively), followed by the *Prevotella* enterotype (16.1% and 21.4% for survivors and nonsurvivors, respectively). Mean relative abundances of all genera in survivors versus nonsurvivors are shown in Supplementary Table 2.

**Fig. 3:**
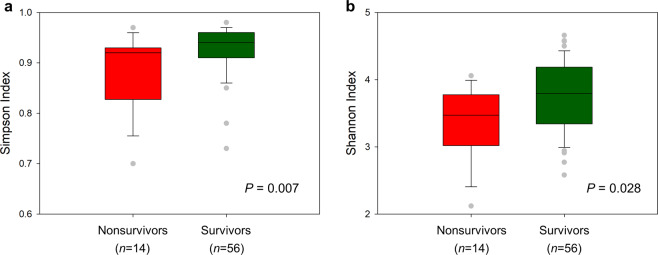
Comparison of different metrics of α-diversity between nonsurvivors and survivors after matching. **a** Simpson index, **b** Shannon index. *P* values were obtained using the Mann–Whitney U test.

**Fig. 4:**
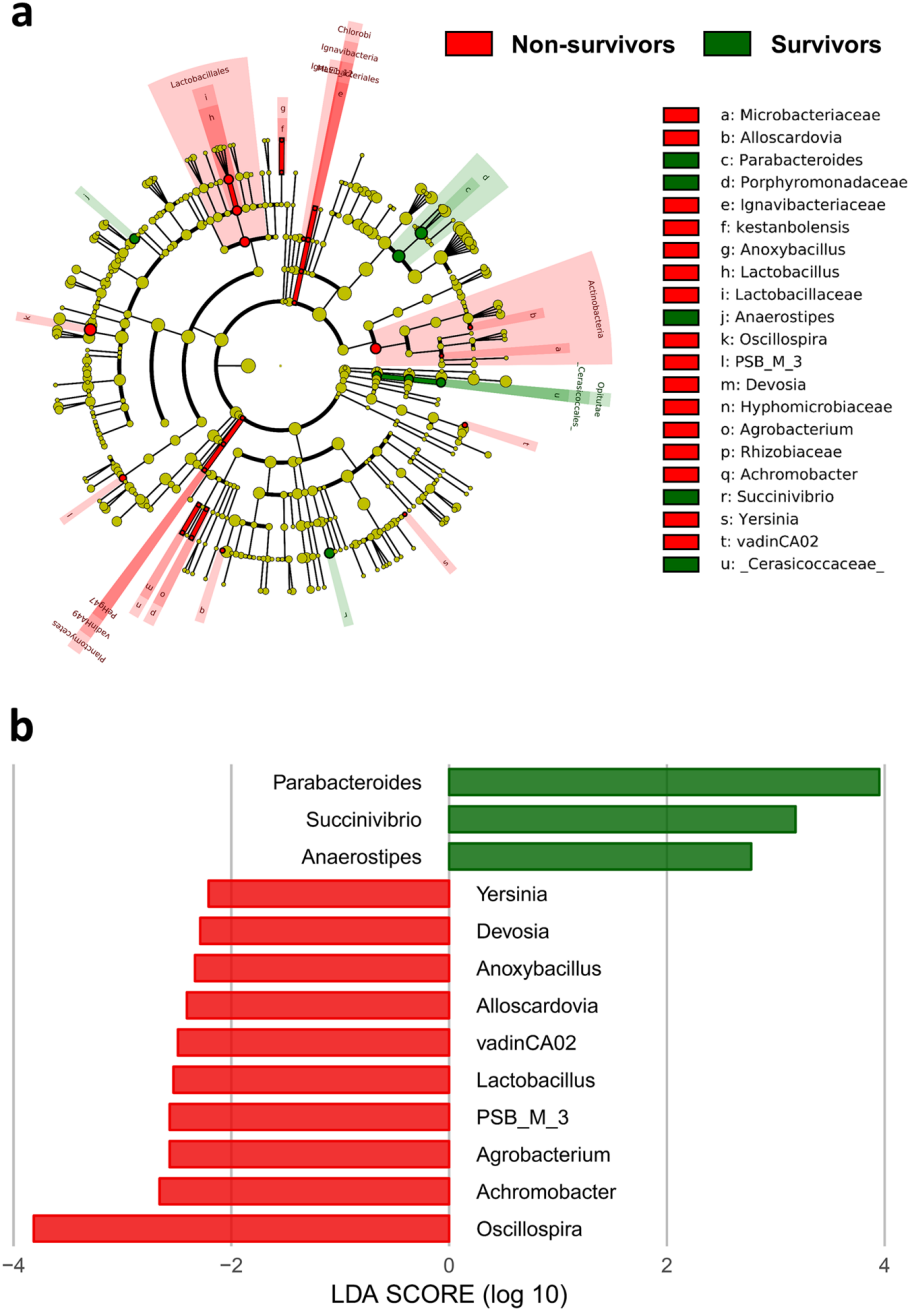
Cladogram showing differentially abundant taxa of the gut microbiota. **a** Differential taxon features at the genus level identified by LEfSe according to nonsurvivors and survivors after matching (LDA score >2). **b** Red and green bars represent taxon features with significantly higher expression in nonsurvivors and survivors, respectively. LDA linear discriminant analysis, LEfSe linear discriminant analysis effect size.

**Fig. 5:**
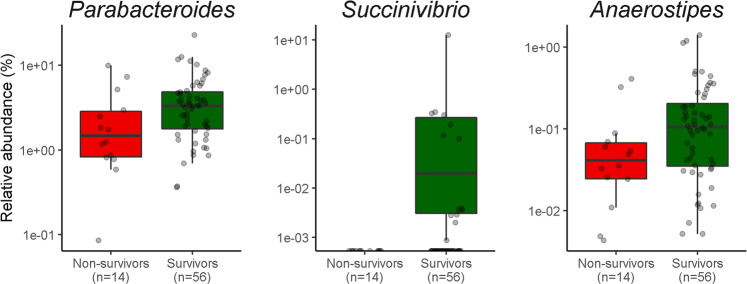
Relative abundance of the gut microbiota at the genus level between nonsurvivors and survivors after matching. *Parabacteroides*, *Succinivibrio*, and *Anaerostipes* were enriched in survivors compared with nonsurvivors. *P* < 0.05 using the Mann–Whitney U test.

α-Diversity analysis revealed significant differences between groups. Nonsurvivors had significantly lower values for the Simpson index (*P* = 0.007) and the Shannon index (*P* = 0.028) (Fig. 3). The difference in microbial composition (β-diversity) between the two groups was insignificant (*P* = 0.482) (Supplementary Fig. 2). LEfSe analysis showed 32 discriminating taxon features between the two groups across different taxonomic levels (Fig. 4). At the genus level, *Parabacteroides*, *Succinivibrio*, and *Anaerostipes* were enriched in survivors. Their relative abundance with respect to mortality status is depicted in Fig. 5. In contrast, nonsurvivors demonstrated a higher expression of *Oscillospira*, *Achromobacter*, *Agrobacterium*, *PSB_M_3*, *Lactobacillus*, *vadinCA02*, *Alloscardovia*, *Anoxybacillus*, *Devosia*, and *Yersinia*.

## Discussion

CKD, a growing public health concern, substantially increases the risk of mortality^1,2^. We observed that lower gut microbial diversity was associated with higher mortality in patients with ESKD on maintenance hemodialysis. On average, survivors demonstrated higher microbial diversity than nonsurvivors. The observed associations are consistent with the notion that diversity is an important feature of a healthy microbiota^10^. Moreover, our results extend beyond microbial diversity to provide evidence that a distinct gut microbial composition may have a significant role in ESKD patient outcomes. Specifically, we found that the relative abundance of *Succinivibrio* and *Anaerostipes*, two short-chain fatty acid (SCFA)-producing bacteria^15,16^, was markedly reduced in nonsurvivors compared with survivors, suggesting that specific taxonomic configurations of the human gut microbiota may reflect health-associated changes that are linked to increased mortality.

Changes in the normal or healthy composition of the intestinal microbiota, referred to as gut dysbiosis, could be defined as the expansion of pathogens, loss of beneficial microbes, and/or reduced microbial diversity^17^. Decreased gut microbial diversity has been reported in a myriad of both intestinal and extraintestinal disorders, such as inflammatory bowel disease, obesity, hypertension, DM, and CKD^18–22^. Our results corroborate previous findings by showing that patients with ESKD with lower diversity were more likely to have preexisting CVD and higher Charlson comorbidity index scores. Notably, although a more diverse microbial community is often considered to constitute a healthier host–microbiome relationship, recent metabolomics studies have demonstrated that certain gut-derived uremic toxins are strongly and positively correlated with intestinal microbial diversity in cohorts of healthy individuals^23,24^. Thus, large-scale and integrated omics studies are required to expand our understanding of the complex interactions between microbial diversity and host health in the context of CKD.

The association of lower microbial diversity with poor survival may be due, in part, to a higher risk of developing CVD and infectious complications. Gut microbial diversity has been shown to be inversely associated with blood pressure and arterial stiffness^20,25^. Although correlations have not been shown between coronary artery disease and heart failure with gut microbiota, recent studies have identified relevant microbial metabolic pathways consistent across several study cohorts, in particular the production of trimethylamine N-oxide, secondary bile acids, and SCFAs^26^. Gut dysbiosis can also be reasonably presumed to increase the risk of infection. Taur et al.^27^ observed that a decline in intestinal microbial diversity with domination of a single bacterial taxon predicted subsequent bloodstream infections in patients undergoing allogeneic hematopoietic stem cell transplantation. Similar findings have been described by Kato et al.^28^. They showed that loss of microbial diversity was associated with increasing episodes of bloodstream infections among patients undergoing liver transplantation. We observed a significant association of lower microbial diversity with CV events, but not with infection-related hospitalizations. The reasons for the discrepancy of the findings between CVD and infection may be explained by lower incidence of bloodstream infections in the present study.

Inflammation may be the mediator linking gut dysbiosis with adverse outcomes in ESKD. Chronic low-grade inflammation is a hallmark of patients with CKD, but its etiology remains obscure. Our findings of the association between lower microbial diversity and inflammation in ESKD are consistent with the results of previous studies and strongly support the notion that gut dysbiosis is involved in the development of chronic inflammation in CKD^29–31^. Beyond microbial diversity, we identified significant taxon differences between nonsurvivors and survivors, with a higher expression of two SCFA-producing bacteria, *Succinivibrio* and *Anaerostipes*, in the survivor group. SCFAs have been shown to have a wide range of impacts on host physiology, including anti-inflammatory effects and the maintenance of gut integrity^32^. The gut microbiota plays an essential role in the development of the host immune system. The immune system in return has evolved largely to maintain its symbiotic relationship with the highly diverse microbiota^33^. However, the disruption of this homeostasis in the presence of a uremic environment may hamper the anti-inflammatory responses by the gut microbiota.

Our study has several limitations. First, given the nature of observational studies, our results do not support causal relationships. Second, fecal samples were collected at baseline only. However, microbial diversity may decrease over time in patients with ESKD with poor survival and reach a minimum value around the time of an event. Therefore, a higher baseline microbial diversity would only bias the study results toward the null hypothesis. Third, although we excluded patients who had used antibiotics in the 3 months prior to enrollment, the long-term effect of antibiotic administration on gut microbiota could not be excluded^34^. Fourth, we only investigated the correlation between gut microbiota and clinical outcomes in this study. Further research will be required to determine if functional characteristics of the microbiome in CKD may be as important as or more important than the contribution of any specific taxa. Fifth, the sample size and death number of our study were relatively small, precluding further adjustment for relevant covariates in multivariate analyses and the ability to conduct subgroup analyses to investigate the effect of gut microbiota on death from different causes. Finally, the study cohort was older, with longer dialysis vintage, and with higher prevalence of diabetes and CVD than have been reported in other studies^35,36^. Thus, the findings may not be generalizable to the overall hemodialysis population.

In conclusion, in this study, we show that gut microbial diversity and composition are strongly correlated with all-cause mortality in ESKD patients receiving maintenance hemodialysis. Our findings also suggest that inflammation may be involved in the pathogenesis of gut dysbiosis with poor survival. Future studies with larger sample sizes and longer follow-up durations are needed to validate our observations and to investigate whether interventions for specific microbial targets may modify the outcomes for hemodialysis patients.

## Methods

### Study design and patient population

This was a prospective cohort study conducted in the outpatient dialysis unit of Taipei Tzu Chi Hospital, Taiwan. The study design and patients were previously described^13,14^. Briefly, 250 patients with ESKD undergoing hemodialysis three times weekly for at least 3 months were assessed for eligibility for inclusion from November 2017 to February 2018. Patients were excluded if they had active malignancies or liver cirrhosis or had used antibiotics in the 3 months prior to enrollment. Information on participant demographics and comorbidities was obtained from interviews and medical record reviews at the time of enrollment. DM was defined by self-reported history or use of oral antidiabetic agents or insulin. The presence of CVD was defined as coronary artery disease, as documented by coronary angiography or a history of myocardial infarction, class III or IV congestive heart failure, or a cerebrovascular accident. This study was conducted in accordance with the Declaration of Helsinki and was approved by the institutional review board of Taipei Tzu Chi Hospital (07-X01-002). All participants provided written informed consent.

### Clinical data collection

Anthropometry and body composition measurements were performed 1 h after the mid-week hemodialysis session by trained nursing staff using standardized procedures. Body weight and height were measured with participants wearing indoor clothing without shoes using an autoanthropometer (Seca, Hamburg, Germany). BMI was calculated as weight in kilograms divided by height in meters squared (kg/m^2^). Body composition was assessed using a portable whole-body bioimpedance spectroscopy device, the body composition monitor (BCM, Fresenius Medical Care, Bad Homburg, Germany). The use of the BCM has been validated among healthy controls from the same ethnic background as the study population^37^. Lean tissue mass and fat tissue mass (fat mass and adipose water) based on a three-compartment model were derived from the impedance data and were expressed as the lean tissue index (lean tissue mass/height^2^) and fat tissue index (adipose tissue mass/height^2^), respectively^38^. Dietary data were collected using a modified short-form food frequency questionnaire^39^. Nutritional status was assessed with the 7-point SGA^40^. Both dietary and nutritional assessments were conducted by a registered dietitian. Physical activity was assessed by the PASE score, which has been validated in ESKD^41^.

### Laboratory measurements

Blood was drawn after an 8-h fasting period, immediately before dialysis during the mid-week dialysis session. The serum albumin concentration was measured using the bromocresol green method. Plasma levels of IL-6 and TNF-α were measured using commercially available ELISA kits based on the manufacturer’s instructions (R&D Systems, Minneapolis, MN). Other laboratory measurements, including glucose, lipids, and electrolytes, were determined according to routine laboratory methods.

### Outcome data collection

Mortality data including the cause of death were ascertained from official death certificates. Patients were censored at the time of transferring to other hemodialysis units, receiving kidney transplantation, or at the end of follow-up in February 2020.

### Fecal sample collection

Fecal samples were obtained at home using a specimen collection kit and delivered to the laboratory (Germark Biotechnology, Taichung, Taiwan) within 24 h by refrigerated (4 °C) transportation. The samples were subsequently aliquoted, and a 200-mg subsample was immediately kept in InhibitEx buffer (Qiagen, Valencia, CA). DNA was extracted using the Qiagen DNA Mini Kit (Qiagen, Valencia, CA). The bacterial DNA concentration was measured with a NanoDrop ND-1000 (Thermo Scientific, Wilmington, DE).

### 16S ribosomal RNA gene sequencing and data processing

Amplification of genomic DNA was performed using bar-coded primers (341F and 805R) that targeted the V3–V4 regions of the bacterial 16S rRNA gene^42^. A paired-end library (insert size of 465 bp for each sample) was constructed with the TruSeq Nano DNA Library Prep kit (Illumina, San Diego, CA). Amplicons were sequenced on an Illumina MiSeq 2000 sequencer using a MiSeq Reagent Kit v3 (Illumina). To minimize batch effects, all samples were sequenced at the same time in the same research laboratory (Germark Biotechnology, Taichung, Taiwan). On a per-sample basis, paired-end reads were merged using USEARCH (v8.0.1623), setting 8 bp as the minimum overlap of read pairs^43^. Merged sequences were quality trimmed using Mothur (v1.35.1). Those reads that did not meet the quality criteria of a minimum quality score of 27 and sequence length shorter than 400 bp or longer than 550 bp for 16S amplicon reads were removed^44^. Chimeric sequences were identified and deleted by USEARCH (reference mode and 3% minimum divergence). Clustering of sequence reads into operational taxonomical units (OTUs) at 97% identity level was achieved using the UPARSE pipeline, and identified taxonomy was then aligned using the Greengenes reference database^45,46^.

### Bioinformatic analyses

α-Diversity, a measure of the richness and evenness of taxa within each sample, was estimated by calculating the Simpson and Shannon indices with the R package phyloseq^47^. The Simpson and Shannon indices take into account the number of species present, as well as the relative abundance of each species, in a single indicator. β-Diversity, comparing the microbial community structures between groups, was calculated based on the Bray–Curtis distance matrices and displayed using principal coordinates analysis (PCoA) by the R package ade4, and the between-group inertia percentages were tested using the Monte-Carlo test with 1000 permutations to determine the *P* value of the ordination results^48^. Both the α-diversity and β-diversity were calculated at the OTU level without prior rarefaction^49^. OTU differences between groups were obtained using LEfSe, which uses the Kruskal–Wallis and Wilcoxon–Mann–Whitney tests to identify taxon features that differ in abundance between groups. Only taxa with an LDA score >2 and a significance of *α* < 0.05 were presented^50^. The results were plotted in a cladogram according to their phylogenetic relationship. The reference-based enterotype is predicted by the classifier trained from 278 MetaHIT samples^51,52^.

### Statistical analyses

Categorical data are presented as frequencies and percentages and were compared by the chi-square test and Bonferroni post hoc test. Continuous data with or without a normal distribution are presented as the means ± standard deviations or medians and interquartile ranges and were compared by Student’s *t* test or the Mann–Whitney U test, respectively. Univariate correlations between the α-diversity and clinically relevant variables were assessed by Spearman’s correlation analyses. Because there is a lack of a definite cut-off value for α-diversity, we divided the patients into two groups according to the median diversity value calculated by using the Simpson index for comparing the mortality risk. The association between α-diversity and all-cause mortality was examined by using the Kaplan–Meier method and log-rank test. Cox proportional hazards modeling was applied to estimate the risk of death. The proportional hazards assumption was visually inspected by log–log survival curves. Because the event rate was relatively low, we avoided overfitting the model by selecting three clinically relevant covariates (age, sex, and Charlson comorbidity index) in adjusted models. Two-tailed *P* values <0.05 were considered statistically significant. All statistical analyses were carried out using the Statistical Package for the Social Sciences software, version 20.0 (SPSS Inc., Chicago, IL).

### Reporting summary

Further information on experimental design is available in the Nature Research Reporting Summary linked to this article.

## Supplementary information

Supplementary Information

Reporting Summary

## Data Availability

The datasets generated for this study can be found in NCBI with accession code PRJNA694038 (https://www.ncbi.nlm.nih.gov/bioproject/PRJNA694038).

## Code availability

All code used to perform the analyses and generate the corresponding figures are publicly available at the Dryad Digital Repository (10.5061/dryad.k3j9kd55d).
