# Supplementary material for: Gut dysbiosis and mortality in hemodialysis patients
Source: NPJ Biofilms Microbiomes. 2021 Mar 3;7:20. doi: 10.1038/s41522-021-00191-x (PMC7930281; doi:10.1038/s41522-021-00191-x)

Supplementary information: Lin et al.

Gut dysbiosis and mortality in hemodialysis patients

Supplementary Tables 1 and 2

Supplementary Figures 1 and 2

Supplementary Table 1. Multivariable Cox regression analysis for the relative risk of cardiovascular event and infection-related hospitalization calculated for a Simpson index below or above the median

	Unadjusted<br>HR (95% CI)	Model 1<br>HR (95% CI)	Model 2<br>HR (95% CI)
Simpson index by median			
Cardiovascular events			
Lower	1.0	1.0	1.0
Higher	0.35 (0.15–0.84)	0.36 (0.15–0.87)	0.36 (0.15–0.88)
P value	0.019	0.023	0.026
Infections			
Lower	1.0	1.0	1.0
Higher	0.65 (0.31–1.37)	0.68 (0.32–1.45)	0.71 (0.33–1.52)
P value	0.255	0.318	0.374

Abbreviations: CI, confidence interval; HR, hazard ratio.

Model 1 was adjusted for age and sex.

Model 2 was adjusted for age, sex, and the Charlson comorbidity index.

Supplementary Table 2. Mean relative abundances of all genera in survivors versus nonsurvivors

Genera	Mean value of relative abundance		*P* value	Multiple testing adjusted *P* value
	Survivors	Nonsurvivors		
Succinivibrio	353.75	0	0.040415962	0.491891421
Bifidobacterium	74.60714286	351.9285714	0.059892829	0.519081981
Fusobacterium	2268.625	667.8571429	0.449516799	0.830475172
Haemophilus	149.9464286	52.42857143	0.385936351	0.830475172
cc_115	3.214285714	5.214285714	0.001403126	0.371828276
Anaerostipes	169.3392857	70	0.107853759	0.714678225
Oscillospira	1383.803571	2855.214286	0.007054513	0.491891421
Cetobacterium	213.625	19.92857143	0.51634129	0.830475172
Methanobrevibacter	3	185.7142857	0.036403811	0.491891421
Blautia	296.5535714	604.4285714	0.041260632	0.491891421
Roseburia	311.25	228.1428571	0.697169792	0.843676427
Desulfovibrio	359.5892857	219	0.988283954	1
Salmonella	4.464285714	2	0.446609307	0.830475172
Acidaminococcus	114.5714286	7.071428571	0.477504166	0.830475172
Megamonas	12.41071429	8.928571429	0.697226934	0.843676427
Coprobacillus	6.142857143	25	0.032150447	0.491891421
Paraprevotella	174.8035714	75.14285714	0.923948491	0.967772135
Citrobacter	38.76785714	5.428571429	0.161602341	0.830475172
Aggregatibacter	2.571428571	0.285714286	0.092097117	0.706291781
Actinobacillus	5.607142857	7.428571429	0.566971435	0.830475172
Actinomyces	0.785714286	3.357142857	0.273912033	0.830475172
Megasphaera	15.46428571	109.8571429	0.904853409	0.959144614
Sutterella	3977.660714	1850.071429	0.592016983	0.830475172
Parabacteroides	3422.196429	2504.285714	0.169806905	0.830475172
Neisseria	4.232142857	0.642857143	0.270492514	0.830475172
Odoribacter	767.6428571	247	0.343586168	0.830475172
Anaerotruncus	11.17857143	19.64285714	0.010233111	0.491891421
WAL_1855D	1.071428571	0.071428571	0.105454967	0.714678225
Streptococcus	67.78571429	136.3571429	0.114322718	0.721321911
Turicibacter	11.96428571	3.928571429	0.261637225	0.830475172
Dehalobacterium	4.75	5.214285714	0.969466189	0.995769535

Cloacibacillus	3.25	44.14285714	0.315338912	0.830475172
Butyricimonas	392.6428571	238.2142857	0.976572893	0.999196203
Ruminococcus	444.1071429	276.9285714	0.523000357	0.830475172
Ruminococcus	2889.589286	1747	0.576882448	0.830475172
Catenibacterium	115.875	2.142857143	0.242061515	0.830475172
Granulicatella	1.821428571	3.5	0.515178857	0.830475172
Eggerthella	2.5	5.285714286	0.016524273	0.491891421
CF231	0.75	0	0.14018209	0.790388378
Methanosphaera	0.053571429	0	0.492223797	0.830475172
vadinCA11	2.571428571	0	0.391031577	0.830475172
Corynebacterium	0.071428571	0	0.315274396	0.830475172
Rothia	1.339285714	0.785714286	0.875217436	0.940654755
Gardnerella	0.107142857	0	0.492223797	0.830475172
Scardovia	0.053571429	0.357142857	0.236289165	0.830475172
Adlercreutzia	4.785714286	15.85714286	0.915110809	0.962318907
Atopobium	0.107142857	1.214285714	0.079385889	0.637492741
Collinsella	99.875	142.7142857	0.458198769	0.830475172
Slackia	1.160714286	0.5	0.535168686	0.830475172
Prevotella	161.7857143	125.5	0.718047853	0.857129194
Prevotella	5790.125	9704.714286	0.814266205	0.895354956
YRC22	0.392857143	0.071428571	1	1
5_7N15	0.017857143	0	0.642443058	0.830475172
Bacteroides	32740.89286	38861.28571	0.947320687	0.984470518
Dysgonomonas	0.589285714	0.214285714	0.779497541	0.895354956
Paludibacter	0.089285714	0.071428571	0.843977256	0.92038672
Porphyromonas	3.071428571	0.642857143	0.796695625	0.895354956
Tannerella	0.035714286	0	0.492178333	0.830475172
Blvii28	0.035714286	0	0.492178333	0.830475172
Capnocytophaga	0.375	0.071428571	0.600988187	0.830475172
Anoxybacillus	0	0.071428571	0.049496967	0.491891421
Eubacterium	77.91071429	51.07142857	0.638054848	0.830475172
Staphylococcus	0.125	0.071428571	0.584964912	0.830475172
Gemella	0.25	0	0.20934059	0.830475172
Enterococcus	6.035714286	18	0.419380601	0.830475172
Sneathia	1.732142857	0	0.140147988	0.790388378
Lactobacillus	61.625	85.21428571	0.01078143	0.491891421
Holdemania	4.839285714	4.142857143	0.34239756	0.830475172
Leuconostoc	0.107142857	0.857142857	0.471893708	0.830475172

Lachnospira	513.5178571	258.2857143	0.628000372	0.830475172
Lactococcus	0.142857143	0.5	0.033213503	0.491891421
Anaerofustis	0.517857143	1.571428571	0.21882568	0.830475172
Fusibacter	0.017857143	0	0.642443058	0.830475172
Veillonella	413.8392857	187.6428571	0.390058015	0.830475172
Oxalobacter	2.821428571	2.5	0.804141929	0.895354956
1_68	0.125	0.071428571	0.692632784	0.843676427
Coprococcus	158.9285714	177.5714286	1	1
Finegoldia	0.053571429	0.071428571	0.812377362	0.895354956
Leptotrichia	1.392857143	0.071428571	0.448740549	0.830475172
Parvimonas	0.089285714	0.571428571	0.154648303	0.830475172
SMB53	3.285714286	1.857142857	0.800949858	0.895354956
Peptoniphilus	0.125	0.214285714	0.553499308	0.830475172
Dorea	529.75	616.6428571	0.808571338	0.895354956
Sedimentibacter	0.357142857	0	0.256577187	0.830475172
Lautropia	1.142857143	0.357142857	0.672815986	0.843676427
Clostridium	336.1428571	57.28571429	0.964838897	0.994872792
Faecalibacterium	2498.678571	3123.928571	0.332514714	0.830475172
Christensenella	2.910714286	3.428571429	0.667134977	0.843676427
02d06	0.267857143	0	0.20934059	0.830475172
Pseudoramibacter_Eubacterium	0.660714286	3.071428571	0.218440931	0.830475172
rc4_4	1.553571429	0.571428571	0.784403345	0.895354956
Sarcina	0.25	0	0.114285178	0.721321911
Lachnobacterium	59.125	42.28571429	0.903244108	0.959144614
Oribacterium	0.607142857	0.071428571	0.378089412	0.830475172
Lutispora	0.071428571	0.071428571	1	1
Dialister	56.85714286	60.85714286	0.332959575	0.830475172
Phascolarctobacterium	1672.946429	2020.642857	0.449556459	0.830475172
Comamonas	1.303571429	0.214285714	0.649022966	0.834908184
Epulopiscium	0.428571429	0.428571429	0.481973782	0.830475172
Janthinobacterium	0.25	0.5	0.3003916	0.830475172
Selenomonas	0.232142857	0.071428571	0.986879811	1
Allobaculum	0.267857143	0	0.492223797	0.830475172
Moryella	0.053571429	0	0.390930586	0.830475172
Pseudobutyrivibrio	0.142857143	0	0.139249117	0.790388378
Paracoccus	0.214285714	0	0.315509964	0.830475172
Shuttleworthia	0.071428571	0	0.315274396	0.830475172
Peptostreptococcus	1.803571429	0.071428571	0.661957222	0.84335896

Desulfotomaculum	0.053571429	0	0.390930586	0.830475172
Peptococcus	0.071428571	0.071428571	0.308941233	0.830475172
Filifactor	0.035714286	0	0.492178333	0.830475172
Mitsuokella	0.107142857	0	0.391031577	0.830475172
Syntrophomonas	0.053571429	0	0.492223797	0.830475172
p_75_a5	0.053571429	0	0.492223797	0.830475172
Sphingomonas	0.071428571	0	0.492223797	0.830475172
Delftia	0.053571429	0	0.390930586	0.830475172
Bulleidia	0.053571429	0	0.390930586	0.830475172
Eikenella	0.053571429	0	0.390930586	0.830475172
Phyllobacterium	0.053571429	0.071428571	0.308941233	0.830475172
Kingella	0.035714286	0	0.492178333	0.830475172
Bilophila	210.1964286	223.0714286	0.741102631	0.876750881
Campylobacter	3.75	1.071428571	0.2794707	0.830475172
Helicobacter	0.053571429	0	0.492223797	0.830475172
Cardiobacterium	0.089285714	0	0.315431467	0.830475172
Edwardsiella	0.017857143	0	0.642443058	0.830475172
Enterobacter	0.428571429	0.357142857	0.95912054	0.992839622
Erwinia	4.071428571	1.857142857	0.412014167	0.830475172
Klebsiella	231.9821429	507	0.860002917	0.930207237
Morganella	1.321428571	0.285714286	0.278030637	0.830475172
Proteus	2.607142857	1.285714286	0.563918456	0.830475172
Serratia	19.78571429	0.5	0.660099563	0.84335896
Trabulsiella	0.267857143	0.214285714	0.889515304	0.950490144
Acinetobacter	0.071428571	0	0.315274396	0.830475172
Psychrobacter	0.017857143	0.071428571	0.296952065	0.830475172
Pseudomonas	0.053571429	0.071428571	0.812377362	0.895354956
Nevskia	0.017857143	0	0.642443058	0.830475172
Treponema	0.035714286	0	0.642443058	0.830475172
Pyramidobacter	1.678571429	2.142857143	0.713306207	0.855321923
TG5	0.089285714	0	0.391031577	0.830475172
Synergistes	8.267857143	508.2142857	0.811505782	0.895354956
Akkermansia	937.7678571	2112.285714	0.076562167	0.634030448

Supplementary Figure 1. Gut microbiota composition in higher and lower microbial diversity groups at phylum and genus levels.

Phylum level

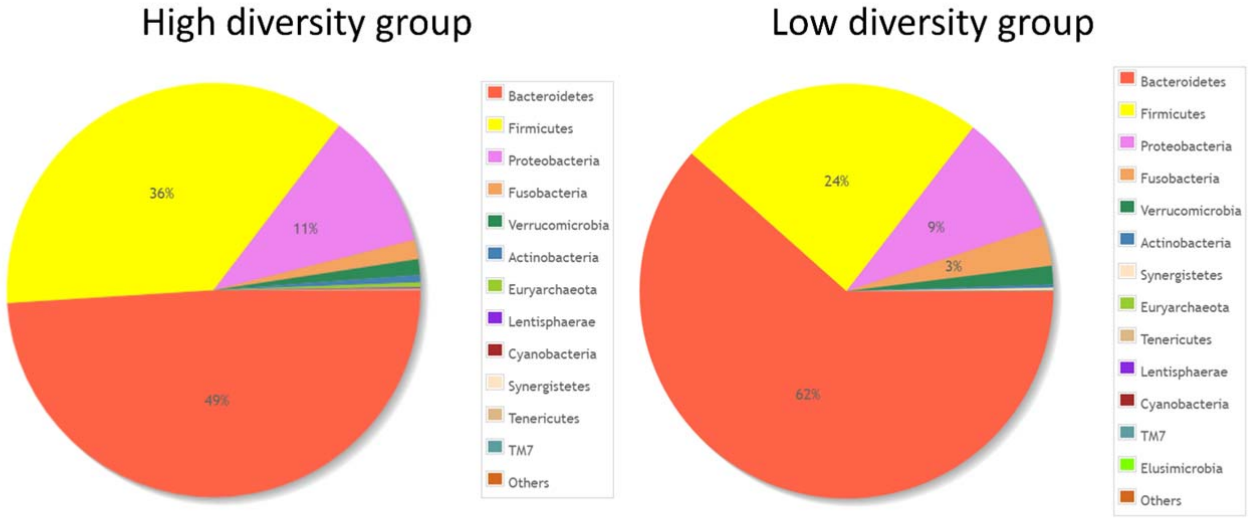

Genus level

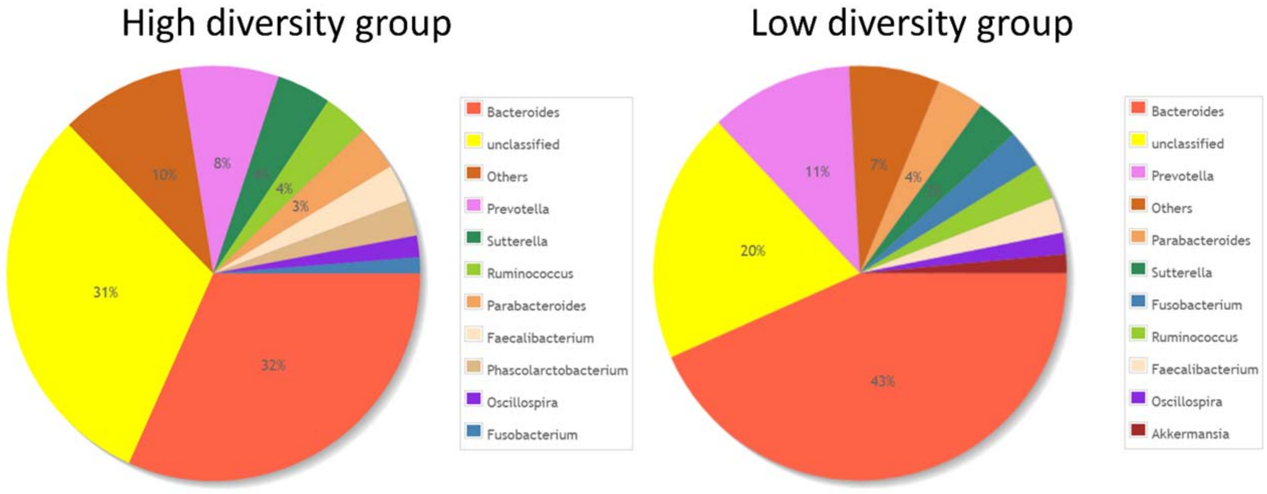

Supplementary Figure 2. Principal coordinate analysis plot of the gut microbiota between the survivors and nonsurvivors based on the Bray-Curtis distances.

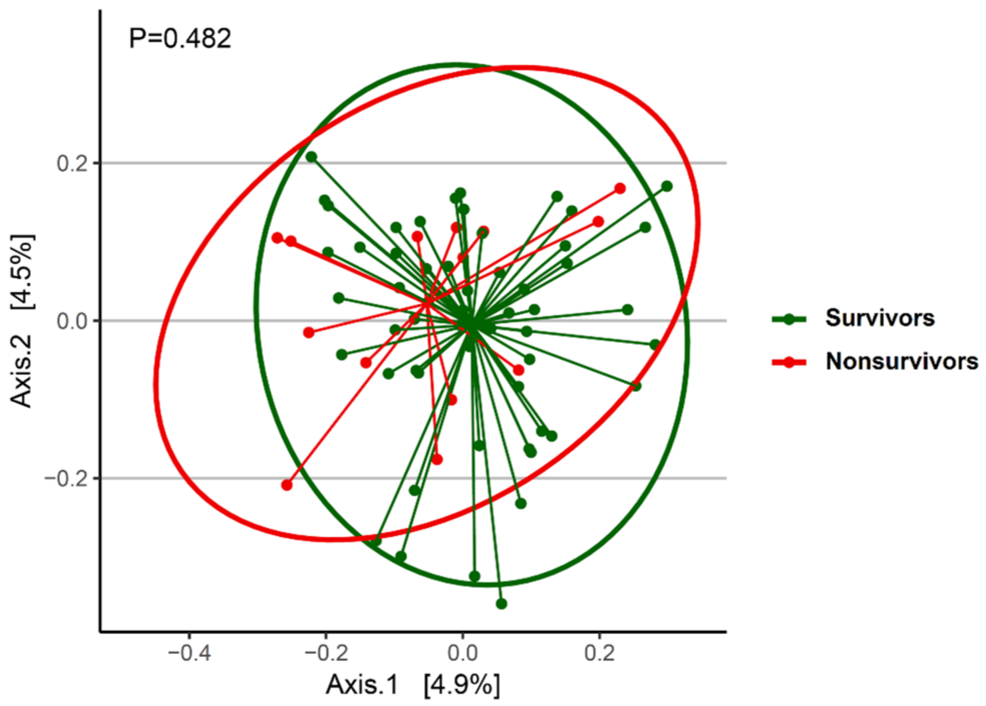

Supplement: Supplementary file 1 — Supplementary Information [file 41522_2021_191_MOESM1_ESM.pdf]
