# Supplementary material for: Gut dysbiosis and mortality in hemodialysis patients
Source: NPJ Biofilms Microbiomes. 2021 Mar 3;7:20. doi: 10.1038/s41522-021-00191-x (PMC7930281; doi:10.1038/s41522-021-00191-x)
Supplement: Supplementary file 2 — Reporting Summary [file 41522_2021_191_MOESM2_ESM.pdf]

nature research

Corresponding author(s): Szu-Chun Hung

Last updated by author(s): Oct 30, 2020

Reporting Summary

Nature Research wishes to improve the reproducibility of the work that we publish. This form provides structure for consistency and transparency in reporting. For further information on Nature Research policies, see our Editorial Policies and the Editorial Policy Checklist.

Statistics

For all statistical analyses, confirm that the following items are present in the figure legend, table legend, main text, or Methods section.

n/a	Confirmed	
☐	☒	The exact sample size (*n*) for each experimental group/condition, given as a discrete number and unit of measurement
☒	☐	A statement on whether measurements were taken from distinct samples or whether the same sample was measured repeatedly
☐	☒	The statistical test(s) used AND whether they are one- or two-sided<br>*Only common tests should be described solely by name; describe more complex techniques in the Methods section.*
☐	☒	A description of all covariates tested
☐	☒	A description of any assumptions or corrections, such as tests of normality and adjustment for multiple comparisons
☐	☒	A full description of the statistical parameters including central tendency (e.g. means) or other basic estimates (e.g. regression coefficient) AND variation (e.g. standard deviation) or associated estimates of uncertainty (e.g. confidence intervals)
☐	☒	For null hypothesis testing, the test statistic (e.g. *F*, *t*, *r*) with confidence intervals, effect sizes, degrees of freedom and *P* value noted<br>*Give P values as exact values whenever suitable.*
☒	☐	For Bayesian analysis, information on the choice of priors and Markov chain Monte Carlo settings
☒	☐	For hierarchical and complex designs, identification of the appropriate level for tests and full reporting of outcomes
☒	☐	Estimates of effect sizes (e.g. Cohen's *d*, Pearson's *r*), indicating how they were calculated

Our web collection on statistics for biologists contains articles on many of the points above.

Software and code

Policy information about availability of computer code

Data collection	the Greengenes reference database
Data analysis	the R package phyloseq; the R package ade4; the Social Sciences software, version 20.0.

For manuscripts utilizing custom algorithms or software that are central to the research but not yet described in published literature, software must be made available to editors and reviewers. We strongly encourage code deposition in a community repository (e.g. GitHub). See the Nature Research guidelines for submitting code & software for further information.

Data

Policy information about availability of data

All manuscripts must include a data availability statement. This statement should provide the following information, where applicable:

- Accession codes, unique identifiers, or web links for publicly available datasets
- A list of figures that have associated raw data
- A description of any restrictions on data availability

We have uploaded the relevant data and code to the Dryad Digital Repository (https://doi.org/10.5061/dryad.k3j9kd55d). Raw data are available upon request.

Field-specific reporting

Please select the one below that is the best fit for your research. If you are not sure, read the appropriate sections before making your selection.

☒ Life sciences ☐ Behavioural & social sciences ☐ Ecological, evolutionary & environmental sciences

For a reference copy of the document with all sections, see nature.com/documents/nr-reporting-summary-flat.pdf

Life sciences study design

All studies must disclose on these points even when the disclosure is negative.

Sample size	Given the exploratory nature of this study, a priori sample size estimation was not performed.
Data exclusions	No data were excluded from this analyses.
Replication	Microbial diversity was significantly different between survivors and nonsurvivors, which can replicated in overall and case-control design.
Randomization	This is an observational study.
Blinding	This is an observational study.

Reporting for specific materials, systems and methods

We require information from authors about some types of materials, experimental systems and methods used in many studies. Here, indicate whether each material, system or method listed is relevant to your study. If you are not sure if a list item applies to your research, read the appropriate section before selecting a response.

Materials & experimental systems

n/a	Involved in the study	
☒	☐	Antibodies
☒	☐	Eukaryotic cell lines
☒	☐	Palaeontology and archaeology
☒	☐	Animals and other organisms
☐	☒	Human research participants
☐	☒	Clinical data
☒	☐	Dual use research of concern

Methods

n/a	Involved in the study	
☒	☐	ChIP-seq
☒	☐	Flow cytometry
☒	☐	MRI-based neuroimaging

Human research participants

Policy information about studies involving human research participants

Population characteristics	Patients with end-stage kidney disease undergoing hemodialysis 3 times weekly for at least 3 months.
Recruitment	250 patients with ESKD undergoing hemodialysis 3 times weekly for at least 3 months in the outpatient dialysis unit of Taipei Tzu Chi Hospital, Taiwan, were assessed for eligibility for inclusion from November 2017 to February 2018. Patients were excluded if they had active malignancies or liver cirrhosis or had used antibiotics in the 3 months prior to enrollment.
Ethics oversight	This study was approved by the institutional review board of Taipei Tzu Chi Hospital (07-X01-002).

Note that full information on the approval of the study protocol must also be provided in the manuscript.

Clinical data

Policy information about clinical studies

All manuscripts should comply with the ICMJE guidelines for publication of clinical research and a completed CONSORT checklist must be included with all submissions.

Clinical trial registration	Not applicable
Study protocol	This is an observational study.
Data collection	250 patients with ESKD undergoing hemodialysis 3 times weekly for at least 3 months in the outpatient dialysis unit of Taipei Tzu Chi Hospital, Taiwan, were assessed for eligibility for inclusion from November 2017 to February 2018. Information on participant

	demographics and comorbidities was obtained from interviews and medical record reviews at the time of enrollment. Anthropometry and body composition measurements, blood testing, and fecal samples collection were collected at baseline.
Outcomes	Mortality data including the cause of death were ascertained from official death certificates. Patients were censored at the time of transferring to other hemodialysis units, receiving kidney transplantation, or at the end of follow-up in February 2020.
